# CHARACTERIZING MUSIC PARTICIPATION FOR ADULTS WITH DOWN SYNDROME

**DOI:** 10.1093/geroni/igae098.2239

**Published:** 2024-12-31

**Authors:** Jennifer Dorris

**Affiliations:** University of Pittsburgh, Pittsburgh, Pennsylvania, United States

## Abstract

Adults with Down syndrome (Ds) are living longer and have high risk of experiencing cognitive decline as they age. Unfortunately, few services are available to support aging adults with Ds and their care partners. Music is a safe, engaging intervention modality that has shown promising results for older adults experiencing cognitive decline. This study reported on adults with Ds’ frequency and type of music participation and described support provided by care partners. Additionally, the study explored associations of age, race, or level of intellectual disability associated with music participation. Care partners of adults with Ds (n =27) completed a survey that measured “listening” and “playing music” using the Guernsey Community Participation and Leisure Assessment. 92.6% of adults with Ds listened to music weekly or daily. 74.1% of care partners listened to music as an unaccompanied activity. 33.3% of adults with Ds played music either weekly or daily, and 48.2% of care partners described playing music as an unaccompanied activity. A significant association was found between race and playing music, but there were no significant associations between age, intellectual disability level, and music participation for adults with Ds. Knowing that racially minoritized adults with Ds had a higher frequency in playing music, those developing services and supports could consider utilizing music as a meaningful activity. Additionally, due to the lack of associations between age and intellectual disability, music programs could be developed equitably for changes in age and intellectual disability level, creating inclusive activities for this growing population of older adults.

